# Tunnel-free acromioclavicular joint reconstruction is associated with improved initial reduction and higher patient satisfaction

**DOI:** 10.1016/j.xrrt.2023.08.002

**Published:** 2023-09-04

**Authors:** Brittany A. Olsen, Joshua W. Rollins, Daniel H. Ngo, James M. Gregory

**Affiliations:** Department of Orthopaedic Surgery, UTHealth Houston McGovern Medical School, Houston, TX, USA

**Keywords:** Acromioclavicular joint, Acromioclavicular reconstruction, Complications, Tunnel-free, Cortical button, Cerclage

## Abstract

**Background:**

Many surgical techniques have been described for acromioclavicular (AC) joint reconstruction. Creation of bone tunnels through the clavicle or coracoid has been shown to be a risk factor for fracture. Use of an AC reconstruction technique that does not create bone tunnels may obviate this risk. This study aims to evaluate clinical outcomes of AC joint reconstruction using a tunnel-free technique compared to reconstruction using a cortical button and clavicular drill holes.

**Methods:**

Consecutive patients who underwent AC joint reconstruction by a single surgeon in a subspecialty referral practice were included. One group of patients received cortical button fixation (button group), in which sutures were passed around the coracoid, brought up through a drill hole in the clavicle, and tied over a cortical button. The other group of patients received tunnel-free fixation, in which a self-locking tape suture was passed in a cerclage fashion around the base of the coracoid and the clavicle and tensioned with a tensioning device (cerclage group). Both groups underwent reconstruction of the coracoclavicular (CC) ligament and AC joint capsule using tibialis anterior allograft. Patient-reported outcome scores and satisfaction were collected and compared between groups. Radiographs were reviewed to evaluate CC ligament distance and loss of reduction.

**Results:**

Twenty-two patients were included in the study (button n = 10, cerclage n = 12). Preoperative demographics and injury characteristics were not different between groups. Average radiographic follow-up was not different between groups (button: 231 days, cerclage: 105 days). Postoperative American Shoulder and Elbow Surgeons, visual analog scale, and Single Assessment Numeric Evaluation scores were similar between groups. Two postoperative clavicle fractures were sustained in the button group. These occurred through clavicular drill holes and were preceded by tunnel widening. No fractures occurred in the cerclage group. CC distance at initial follow-up was significantly less in the cerclage group (button: 11.2 ± 4.5 mm, cerclage: 7.0 ± 2.9 mm, *P* =.023). Loss of reduction was similar throughout the postoperative period (button: 4.3 ± 2.6 mm, cerclage: 4.8 ± 4.1 mm, *P* >.05. Forty percent of patients were unsatisfied with their clavicle after button fixation (n = 4/10), compared with zero after cerclage fixation (n = 0/12, *P* =.03). Reasons for dissatisfaction were fracture (n = 2) and persistent cosmetic deformity (n = 2).

**Conclusion:**

Tunnel-free AC joint reconstruction is associated with improved initial radiographic appearance and patient satisfaction compared to single cortical button fixation. Postoperative clavicle fracture and persistent cosmetic deformity drive patient dissatisfaction, which may be minimized by avoiding clavicular drill holes and using a tensioned self-locking cerclage suture to improve initial reduction.

Injury to the acromioclavicular (AC) joint accounts for approximately 40%-50% of all shoulder injuries.^7^^,^^11^ This injury is frequently encountered and treated by fellowship trained as well as general orthopedic surgeons.^9^^,^^10^ It is widely accepted that Rockwood grade I and II injuries should be treated nonoperatively and that Rockwood grade IV, V, and VI injuries should be treated operatively, but, despite the frequent encounters and extensive research performed on this topic, there is still no consensus on the best management practice of grade III AC joint injuries.^8^^,^^10^ For those injuries that necessitate surgery, more than 60 different surgical techniques have been described and researched.^4^^,^^5^ These include open fixation of the AC and coracoclavicular (CC) joints, Weaver-Dunn and modified Weaver-Dunn, anatomic reconstruction, nonanatomic reconstruction, and arthroscopic-assisted techniques.^4^^,^^5^^,^^8^^,^^10^ All of these operative techniques require fixation and the use of at least one bone tunnel either in the coracoid or the clavicle. The most common complications associated with the variety of techniques include loss of reduction, infection, fracture of the coracoid or clavicle, and the need for either planned or unplanned reoperation.^4^^,^^5^^,^^8^, ^9^, ^10^, ^11^

Initially, we performed AC joint reconstruction using a combination of suspensory fixation through a single clavicular drill hole augmented with allograft augmentation. No coracoid drill holes were used due to reports of coracoid fracture after eccentric coracoid drilling.^2^ After noticing complications associated with the clavicular bone tunnel including tunnel widening and atraumatic clavicle fractures, a new technique was developed that did not require any drill holes in either the coracoid or the clavicle. In a previous publication, we proposed an arthroscopic-assisted, tunnel-free surgical technique in an effort to minimize the risk for subsequent bony failure through fracture while maintaining an adequate reduction using a combination of suspensory and allograft fixation. This technique can be used in both acute and chronic injuries and avoids many common complications seen after surgical management of AC injuries using bone tunnels.^5^

This study aims to evaluate short-term clinical and radiographic outcomes of AC joint reconstruction using the aforementioned tunnel-free technique compared to reconstruction using a cortical button and a single clavicular drill hole, serving as a proof-of-concept. We hypothesized that there would be minimal incidence of operative or postoperative fracture of the acromion or clavicle, maintenance of AC reduction and that clinical outcomes would be similar to the comparative operative technique.

## Materials and methods

After institutional review board approval, a retrospective cohort study was conducted. A total of 22 consecutive patients with unilateral high-grade AC joint injuries who underwent surgical reconstruction were identified through a review of a single surgeon’s billing surgical records. Inclusion criteria for this study included: English-speaking patients greater than 18 years of age who were treated at UTHealth, Department of Orthopedic Surgery for high-grade AC joint injuries (Rockwood grade III or greater) and who received treatment for their injury before October 1, 2021. Patients who presented with polytraumatic injuries, distal clavicle fracture, or any ipsilateral upper extremity injury at the time of the initial injury were excluded. Patients who underwent an isolated CC ligament repair rather than reconstruction were also excluded. Twenty-two patients met inclusion and exclusion criteria and had greater than six months of clinical follow-up.

Once the final list of study patients was obtained, patients’ medical records were used to obtain demographic information, contact information, preoperative clinic notes, date of surgery, intraoperative surgical data, clinic follow-up notes, and radiology images pertaining to the AC joint injury. Patients were then called to answer several surveys to assess clinical outcomes.

Preoperatively, the patient’s initial shoulder pain and function was assessed using the American Shoulder and Elbow Surgeons (ASES) Standardized Shoulder Assessment Form, visual analog scale (VAS) for pain, Simple Shoulder Test, and Brophy Activity Score. We elected to use validated measurements of shoulder function which evaluated performance in activities of daily living, activity level, and pain. Radiographs were obtained to evaluate the CC ligament distance and classify the injury as well as to use as a comparison for postoperatively.

### Surgical technique

**Figure 1:**
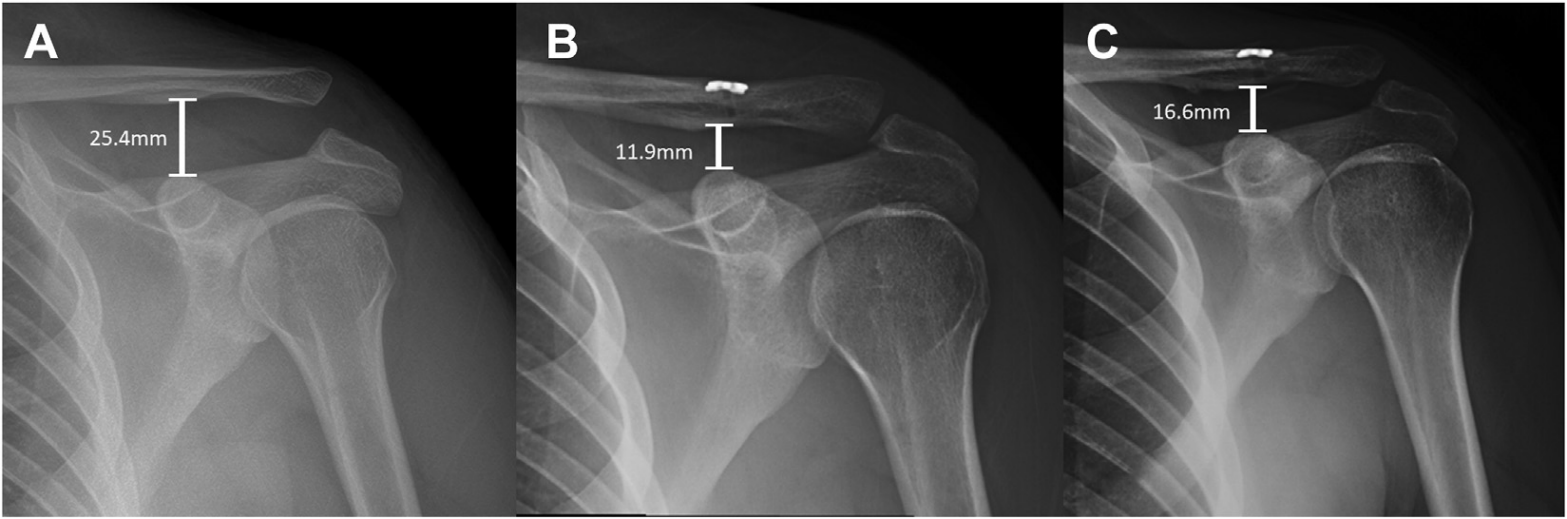
Radiographs of a patient who underwent coracoclavicular reconstruction using suspensory fixation and a clavicular cortical button in conjuction with tibialis anterior allograft (button group). Preoperative radiographs are shown (**A**), followed by initial two-week postoperative radiographs (**B**), and final follow-up at 9 months (**C**). The measured coracoclavicular distance is denoted.

In regards to intraoperative information, there were two groups of patients. One group of patients received cortical button fixation (button group), in which two #5 braided FiberTape (Arthrex, Naples, FL, USA) sutures were passed in a cerclage fashion around the base of the coracoid and brought up through a single 2.5 mm drill hole in the clavicle. The sutures were passed through a cortical button, and the reduction was maintained with the assistant holding a ballspike pusher until the cortical button was seated. This was confirmed fluoroscopically. The sutures were then tied over the top of the cortical button. The fixation was augmented in all patients with a tibialis anterior allograft passed in a cerclage fashion around the coracoid and clavicle, and tied on top of the clavicle. One limb of the allograft was then brought laterally and sutured to the lateral AC joint capsule to reconstruct this disrupted capsular tissue. Representative preoperative and postoperative radiographs are shown in Figure 1.

**Figure 2:**
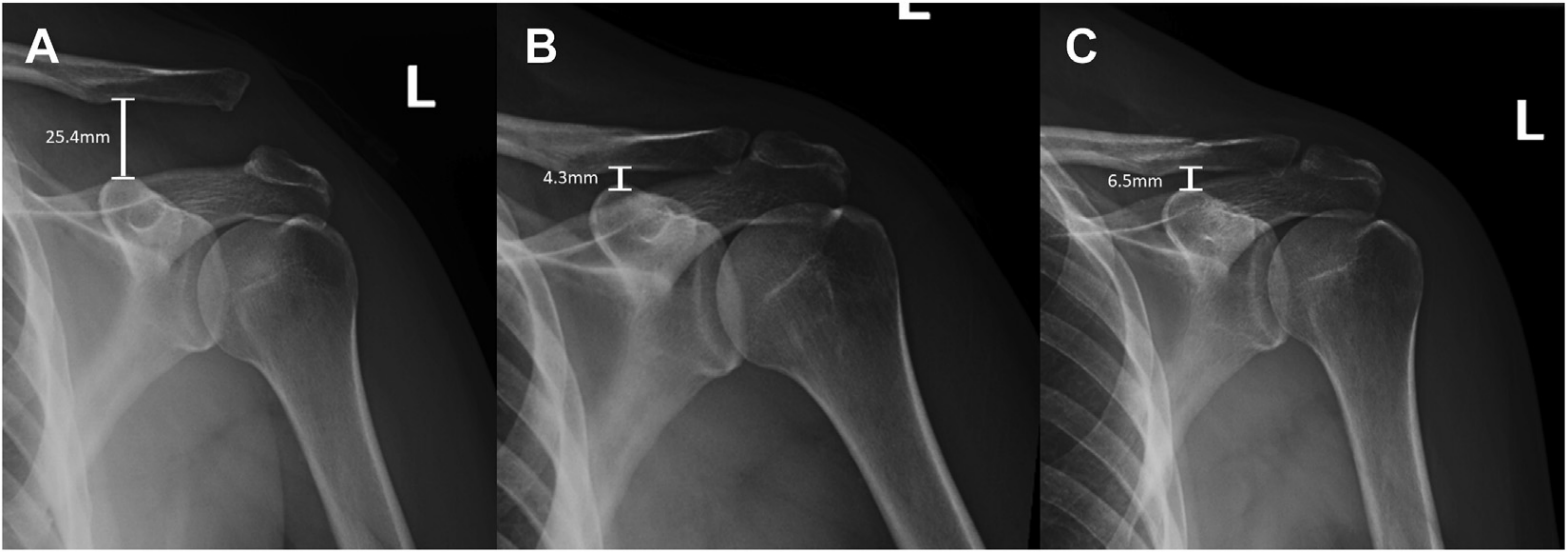
Radiographs of a patient who underwent coracoclavicular reconstruction using suspensory fixation with a tunnel-free technique using a cerclage suture in conjuction with tibialis anterior allograft (cerclage group). Preoperative radiographs are shown (**A**), followed by three-week postoperative radiographs (**B**), and final follow-up at 4 months (**C**). The measured coracoclavicular distance is denoted.

The other group of patients received tunnel-free fixation, in which a braided self-locking tape suture was passed in a cerclage fashion around the base of the coracoid and the clavicle (cerclage group). Once the transition was made to the tunnel-free technique, no further patients received cortical button fixation. Briefly, the tunnel-free technique involves arthroscopic-assisted passage of a #5 FiberWire (Arthrex, Naples, FL, USA) suture around the base of the coracoid to create a cinch stitch around the coracoid, followed by passage of the free tail of a FiberTape Cerclage (Arthrex, Naples, FL, USA) suture from lateral to medial around the base of the coracoid. One tail of the #5 FiberWire and the free limb of the FiberTape Cerclage are passed posteriorly around the clavicle. The reduction was maintained using a ball-spike pusher, and a tensioning device was used to tension the cerclage suture. Once appropriate tension is obtained, the free limbs of the cerclage suture are then tied. After fluoroscopic confirmation of maintenance of reduction, the #5 suture is then tied over the clavicle as well. This fixation was then augmented using a tibialis anterior allograft in an identical fashion as described above. This procedure is further detailed in the technique paper written by Gosselin et al.^5^ Representative preoperative and postoperative radiographs from this technique are shown in Figure 2.

From the surgical data, clinic notes, and radiology images, the following information was recorded: time from injury to surgery, laterality of injury, Rockwood classification, fixation method, follow-up time, complications, initial displacement, intraoperative, reduction, and loss of reduction at the final follow-up.

When obtaining postoperative clinical outcomes, the ASES and VAS were again used. Additionally, the Single Assessment Numeric Evaluation (SANE), the Patient-Reported Outcomes Measurement Information System (PROMIS) Scale Global Health v1.2, the PROMIS Upper Extremity Short Form v2.0, the PROMIS Satisfaction with Social Roles and Activities, the PROMIS Physical Function Short Form 10a, and the Short-Form 36 were utilized to assess clinical outcomes. Radiographs were taken to compare the CC ligament distance and evaluate for loss of reduction postoperatively. We also recorded the patient’s overall satisfaction after undergoing the operation.

Statistical techniques consist of Fisher exact tests for categorical variables. For continuous variables, the Mann-Whitney U test is utilized. Analysis was performed on R-Studio (Boston, MA, USA). Confidence level was 95% indicating that a *P* value less than .05 is significant.

## Results

**Figure 3:**
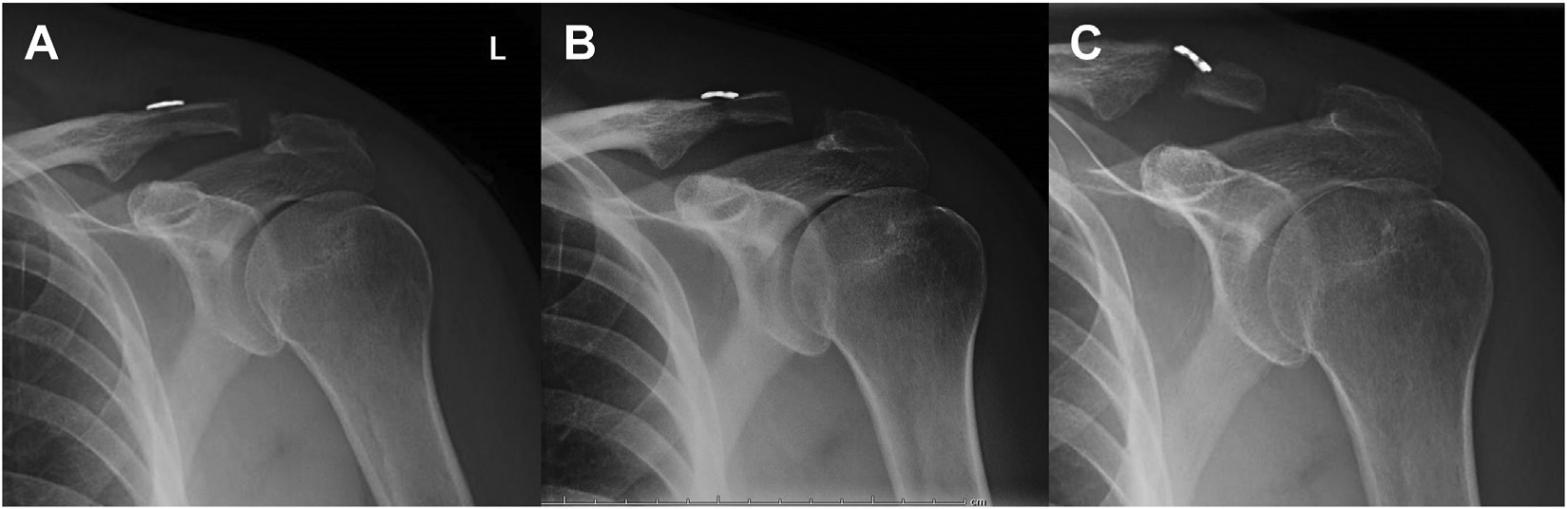
Sequential postoperative radiographs of a right shoulder after acromioclavicular joint reconstruction using a cortical button. Postoperative radiographs are shown at two weeks (**A**), followed by tunnel widening noted at six weeks (**B**), and subsequent clavicle fracture at three months (**C**).

A total of 22 patients were included in the study (button n = 10, cerclage n = 12). Preoperative demographics and injury characteristics were not different between groups (Table I). Average radiographic follow-up was not statistically different between groups (button: 231 days, cerclage: 105 days). Postoperative ASES, VAS, and SANE scores were similar between groups (Table II). Two postoperative low-energy clavicle fractures were sustained in the button group. One patient described bumping into a doorway and the other noted a clavicle deformity with no known preceding trauma. These occurred through clavicular drill holes and were preceded by tunnel widening (Fig. 3). No fractures occurred in the cerclage group. CC distance at initial follow-up was significantly less in the cerclage group (button: 11.2 ± 4.5 mm, cerclage: 7.0 ± 2.9 mm, *P* =.023). Loss of reduction was similar throughout the postoperative period (button: 4.3 ± 2.6 mm, cerclage: 4.8 ± 4.1 mm, *P* >.05), and the cerclage group demonstrated a decreased CC distance at the final follow-up that was not significant (button: 15.5 ± 4.9 mm, cerclage: 11.8 ± 4.7 mm, *P* =.09). Forty percent of patients were unsatisfied with their clavicle after button fixation (n = 4/10), compared with zero percent after cerclage fixation (n = 0/12, *P* =.03). Reasons for dissatisfaction were fracture (n = 2) and persistent cosmetic deformity due to loss of reduction (n = 2).

**Table I:** Preoperative patient demographics and injury characteristics.

	Button (n = 10)	Cerclage (n = 12)	*P* value
Sex			1
Male	9	10	
Female	1	2	
Mean age (y)	39.1	42.5	.74
Mean BMI	27	26.6	.6
Time from injury to surgery (d)	27.3	469	.18
Side			.41
Left	3	6	
Right	7	6	
Classification			1
Type 3	1	1	
Type 4	0	1	
Type 5	9	10	
Preoperative - ASES	32	50.5	.23
Preoperative - VAS	6.1	4.1	.27
Preoperative - Shoulder Test	20	47.2	.41
Initial displacement (%)	189.4 ± 93.8	152.2 ± 54.5	.44

*ASES*, American Shoulder and Elbow Surgeons; *BMI*, body mass index; *VAS*, visual analog scale.

**Table II:** Postoperative patient reported and radiographic outcomes.

	Button (n = 10)	Cerclage (n = 12)	*P* value
Mean radiographic follow-up time (d)	231.8	104.8	.1
Postoperative - ASES	89.3 ± 8.6	87.8 ± 8.5	.67
Postoperative - VAS	1.4 ± 1.1	1.2 ± 1.0	.7
Postoperative - SANE	87.3	91	.53
CC distance at first follow-up (mm)	11.2 ± 4.5	7.0 ± 2.9	.023∗
CC distance at final follow-up (mm)	15.5 ± 4.9	11.8 ± 4.7	.09
Loss of reduction at final follow-up (mm)	4.3 ± 2.6	4.8 ± 4.1	.86
Clavicle fracture	2	0	.19
Patient dissatisfaction	4	0	.03∗

*ASES*, American Shoulder and Elbow Surgeons; *VAS*, visual analog scale; *SANE*, Single Assessment Numeric Evaluation; *CC*, coracoclavicular.

∗ Significant value of *P* < .05.

## Discussion

There have been numerous AC joint reconstruction techniques that have been described and investigated, with no one surgery becoming the gold standard. This is due to the comparable patient-reported outcomes and relatively low complication rates across all the techniques. The overall complication rate of AC joint reconstruction was found to be approximately 21% and the reoperation rate was approximately 7.6%.^1^^,^^4^^,^^5^^,^^8^ The most common complications include loss of reduction in 43% of cases, clavicle fracture in up to 18%, and infection in up to 7% of cases.^1^^,^^4^^,^^5^^,^^8^ In this study, we compared the clinical and radiographic outcomes of AC joint reconstruction using a tunnel-free technique to a reconstruction using a cortical button and clavicular drill holes.

We found that the postoperative patient-reported outcome scores, including the ASES, VAS, and SANE, were similar between groups. This is consistent with many of the other techniques investigated in the literature outside of the 2 techniques that we looked at.^4^^,^^5^^,^^8^ Our study had two postoperative clavicle fractures sustained in the button group while no fractures occurred in the cerclage group. These fractures occurred through clavicular drill holes and were preceded by tunnel widening. We believe that by transitioning to a technique that avoids bone tunnels, we are minimizing the stress risers which may predispose to fracture. With the literature documenting a fracture complication rate of about 18% among all surgical techniques and the button group in our study having a fracture rate of 20%, this appears to be clinically significant.^1^^,^^4^^,^^5^^,^^8^ As seen in our study, this fracture rate impacts the patient’s satisfaction as both of the patients in the button group with a resultant fracture reported being dissatisfied with the outcome of their surgery.

This study also found a significant difference in the initial CC distance with the cerclage group being less than the button group. Throughout the study, it was found that the CC distance in both groups increased roughly the same amount. Therefore, at the final follow-up, the CC distance was less in the cerclage group which can be attributed to the initial reduction and fixation used. Subjectively, we believe that the use of a tensioner with the cerclage technique allows us to gain improved initial reduction at the time of surgery. The fact that our initial CC distance at the first follow-up appointment was significantly lower in the cerclage cohort is likely related to the use of this tensioning device. Both cohorts lost reduction by the time of the final follow-up, but the similarity in the loss of reduction was consistent with prior studies.^6^ However, the cerclage cohort had an average CC distance of 11.8 mm which is regarded as in the normal range.^3^^,^^8^ Initial over-reduction at the time of surgery in the cerclage cohort likely allowed final measurements to settle into this normal range. Clavert et al conducted a prospective multicenter study to evaluate failure rates after reconstruction using a button and found that there were 32 clinical failures and 48 radiographic failures out of the 116 patients studied.^2^ Radiographic failures were described as over 50% loss of reduction at the final follow-up.^4^ In our study, this difference in reduction led to 2 more patients in the button group being dissatisfied with their surgery due to the persistent cosmetic deformity.

One of the limitations of this study is its limited sample size. Although it is possible the study is underpowered to detect certain endpoints, a sample size of 22 patients is comparable to other published studies in the literature and reflects the challenge of large-scale research into AC joint pathology.^8^ Additionally, these data only represent short-term clinical and radiographic outcomes. No long-term data exist comparing these two techniques. One other limitation is that it only compares two techniques in a surgery that has over 60 techniques described.^4^ It is possible that the tunnel widening seen with the single button technique is due to windshield wiping and rotatory motion of the suture being fixated on the clavicle at a single point. A popular variation of a single clavicle button technique includes single drill holes through both the clavicle and the coracoid. However, this still involves a single axis of fixation to the clavicle, while also adding risk for coracoid fracture due to eccentric drilling. Complications with single button clavicle fixation may be obliviated when using a two-tailed technique with multiple drill holes through the clavicle; however, it is out of the scope of this study to compare all variations of surgical technique. Our clinical experience and subsequent patient dissatisfaction with the single button technique led to the change in surgical technique, and so we felt reporting these results would be helpful to other surgeons considering surgical options for AC joint reconstruction.

## Conclusion

Tunnel-free AC joint reconstruction is associated with improved initial radiographic appearance and patient satisfaction compared to single cortical button fixation. Postoperative clavicle fracture and persistent cosmetic deformity drive patient dissatisfaction, which may be minimized by avoiding clavicular drill holes and using a tensioned self-locking cerclage suture to improve initial reduction.

## Disclaimers:

Funding: No funding was disclosed by the authors.

Conflicts of interest: James Gregory discloses Consultant Agreement, Research Support—Stryker; Consultant Agreement, Research Support—Arthrex; Stock/Stock Options—Sparta Biomedical. The other authors, their immediate families, and any research foundation with which they are affiliated have not received any financial payments or other benefits from any commercial entity related to the subject of this article.
